# Supplementary material for: Translocator protein is a marker of activated microglia in rodent models but not human neurodegenerative diseases
Source: Nat Commun. 2023 Aug 28;14:5247. doi: 10.1038/s41467-023-40937-z (PMC10462763; doi:10.1038/s41467-023-40937-z)
Supplement: Supplementary file 1 — Supplementary Information [file 41467_2023_40937_MOESM1_ESM.pdf]

Figure S1 a-i Cytokine secretion of TNF and fold change of IFI44 mRNA of primary mouse and human macrophages and microglia after stimulation, indicating activation of both cell types. In unstimulated cells, TNF concentrations were below the limit of detection (not shown). **i** Representative image of primary human microglia in culture. Biologically independent samples were used for all experiments (**a,b** n=4 for all conditions, **c-h** n=3 for all conditions). Bar graphs mark the mean ± SEM. Source data are provided as a Source Data file.

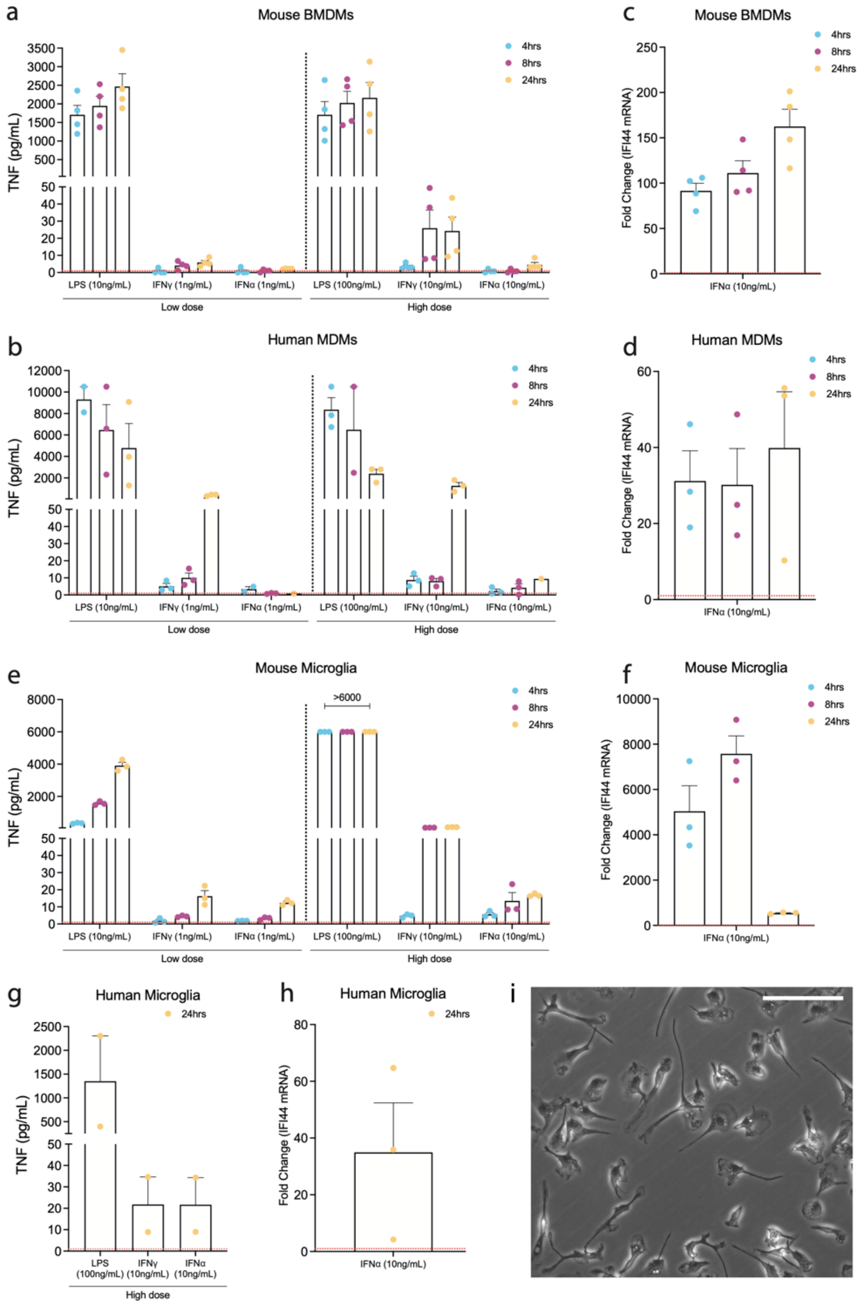

Figure S2. a,b Cytokine secretion of human monocytes from healthy volunteers with C/C or T/T genotype at the rs6971 locus exposed to 100ng/ml LPS for 6h. (two-tailed t-test; TNF: P=0. 957, n = 6 biological replicates; IL6: P=0. 795, n = 7 biological replicates) Box and whiskers mark the 25th to 75th percentiles and min to max values, respectively, with the median indicated. **C** Effect of TSPO genotype (rs6971, C/C vs T/T) on gene expression in human monocytes stimulated with LPS for 24h. There was no difference in macrophage activation genes (GO:0042116) which are marked in red and only 6 genes were differentially regulated (CCL22, MARCKSL1, AC092368.3, TMEM229B, MIR503HG, AC103769.1). Source data are provided as a Source Data file.

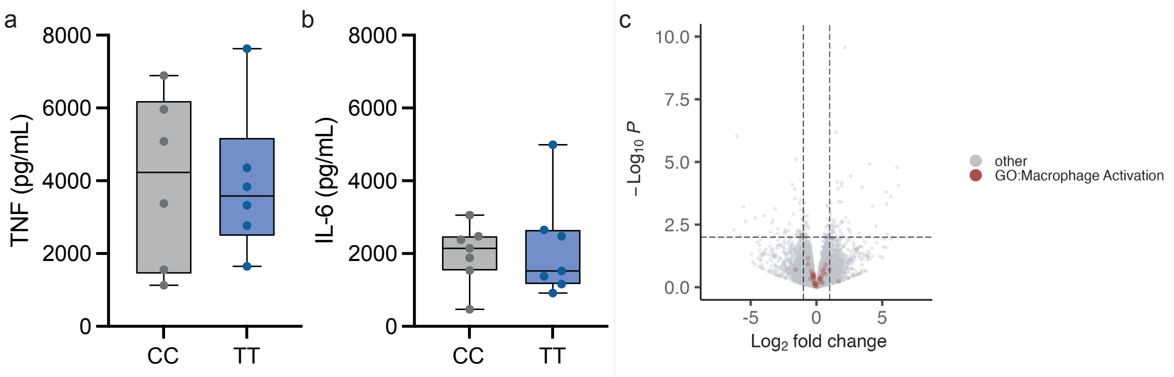

Figure S3. **a** Boxplot showing TSPO fold change in human and mouse macrophages in baseline and IFNγ treated samples. **b** Boxplot showing PU.1 (SPI1) transcription factor and TSPO gene expression change in IFNγ treated macrophages compared to baseline condition. Biologically independent samples were used for all experiments (**a** human: n=3, mouse: n=1 for all conditions) (**b** n=3 for all conditions). Statistical significance was determined using the Wald test. Box and whiskers mark the 25th to 75th percentiles and min to max values, respectively, with the median indicated. Source data are provided as a Source Data file.

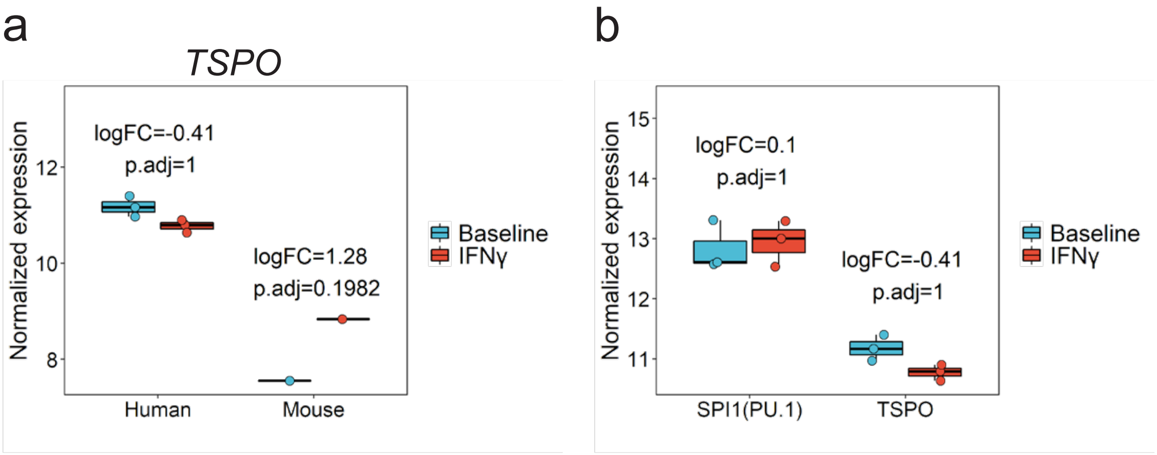

Figure S4. Of the 24 rodent species examined here, 12/24 are from the Muroidea superfamily (purple branches). 10 of these 12 Muroidea species contain the AP1 binding site in the TSPO promoter (Green Highlight). We did not find any rodent species outside the Muroidea superfamily that contain the AP1 binding site in the TSPO promoter. The phylogenetic analysis shows that majority of the species (9/12) from Muroidea superfamily forms a single clade. Phylogenetic tree was generated using the Maximum Parsimony method in MEGA11. The consistency index (CI) is 0.623399 (0.553120) and the retention index (RI) is 0.525671 (0.525671) for all sites and parsimony-informative sites (in parentheses). The percentage of replicate trees in which the associated taxa clustered together in the bootstrap test (1000 replicates) are shown next to the branches. Source data are provided as a Source Data file.

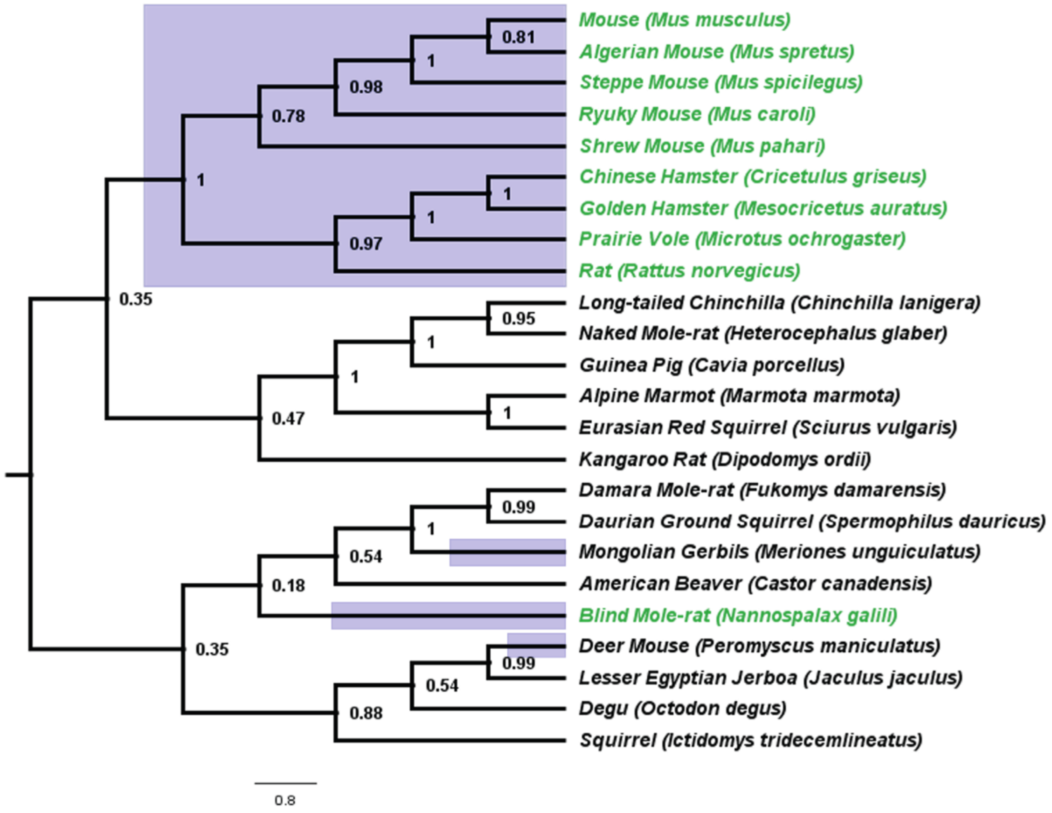

Figure S5. Validation of the TSPO antibody in TSPO$^{-/-}$ mice with different preservation and staining methods as well as with and without the use of Sudan Black to eliminate autofluorescence (scale bar = 50 μm). Source data are provided as a Source Data file.

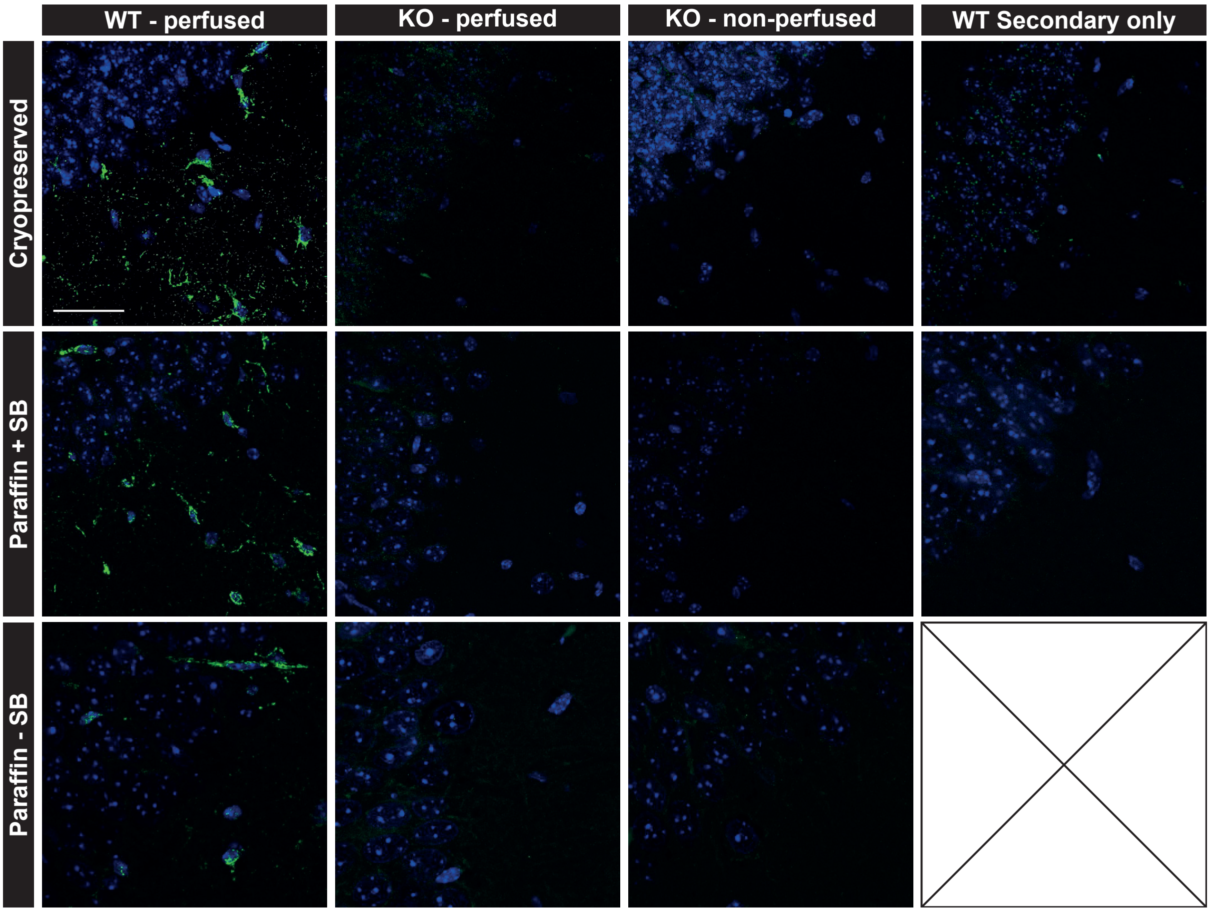

Figure S6. Autoradiography of [^{125}I]CLINDE specific binding in control samples relative to AD samples. **a** Representative brain section labelled with [^{125}I]CLINDE. The white contour delineates the hippocampus. **b** An immediately adjacent brain section in which the non-specific binding of [^{125}I]CLINDE was estimated using an excess of unlabelled compound (10 um). **c** The specific binding of TSPO in the hippocampus (SBR) showed no difference between the two groups (NDC, Braak 0-2 and AD, Braak 5-6, P=0.674). Biologically independent samples were used for all experiments (**b** n=8 NDC and n=8 AD). Statistical analysis was performed by fitting a linear model using the Braak stage as the comparator and the TSPO as a confounder and performing a two-way ANOVA. Bar graphs mark the mean ± SD. Source data are provided as a Source Data file.

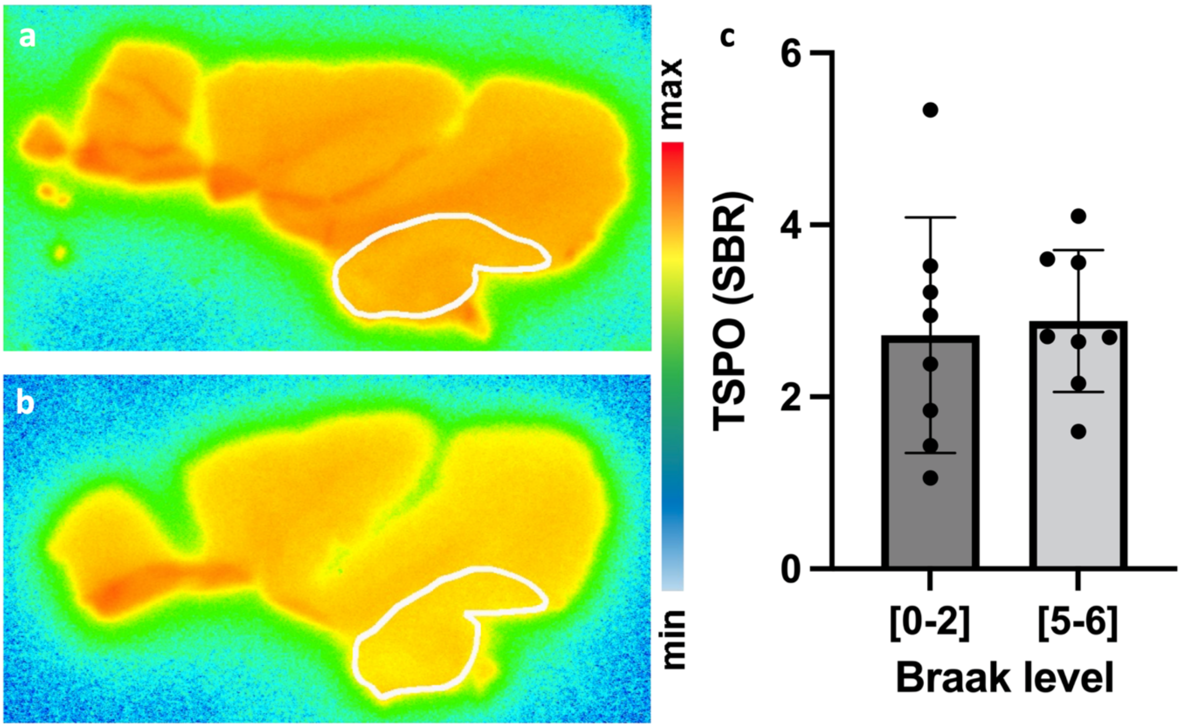

Figure S7. Western blotting quantification of TSPO density in the hippocampus of AD and NDC. The binding of TSPO showed no difference between the two groups using a two-tailed Mann-Whitney test (P=0.5303). Bar graphs mark the mean ±SD. Biologically independent samples were used for all experiments (n=7 NDC and n=5 AD). Source data are provided as a Source Data file.

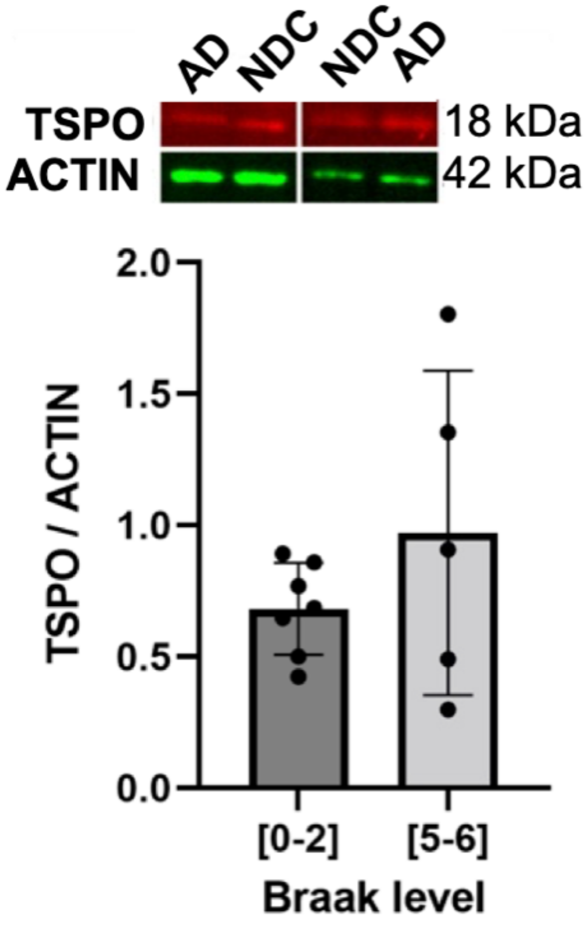

Figure S8. Individual IMC data of TSPO density near plaques and neurofibrillary tangles in the AD brain. IMC results comparing TSPO, CD68 and HLA-DR density in IBA1+ cells with respect to their proximity to (a) amyloid plaques and (b) NFT. Data points corresponding to cells in Figure 4 are replaced here by data points corresponding to individual subjects. Dot plots mark the mean ± SEM. Source data are provided as a Source Data file.

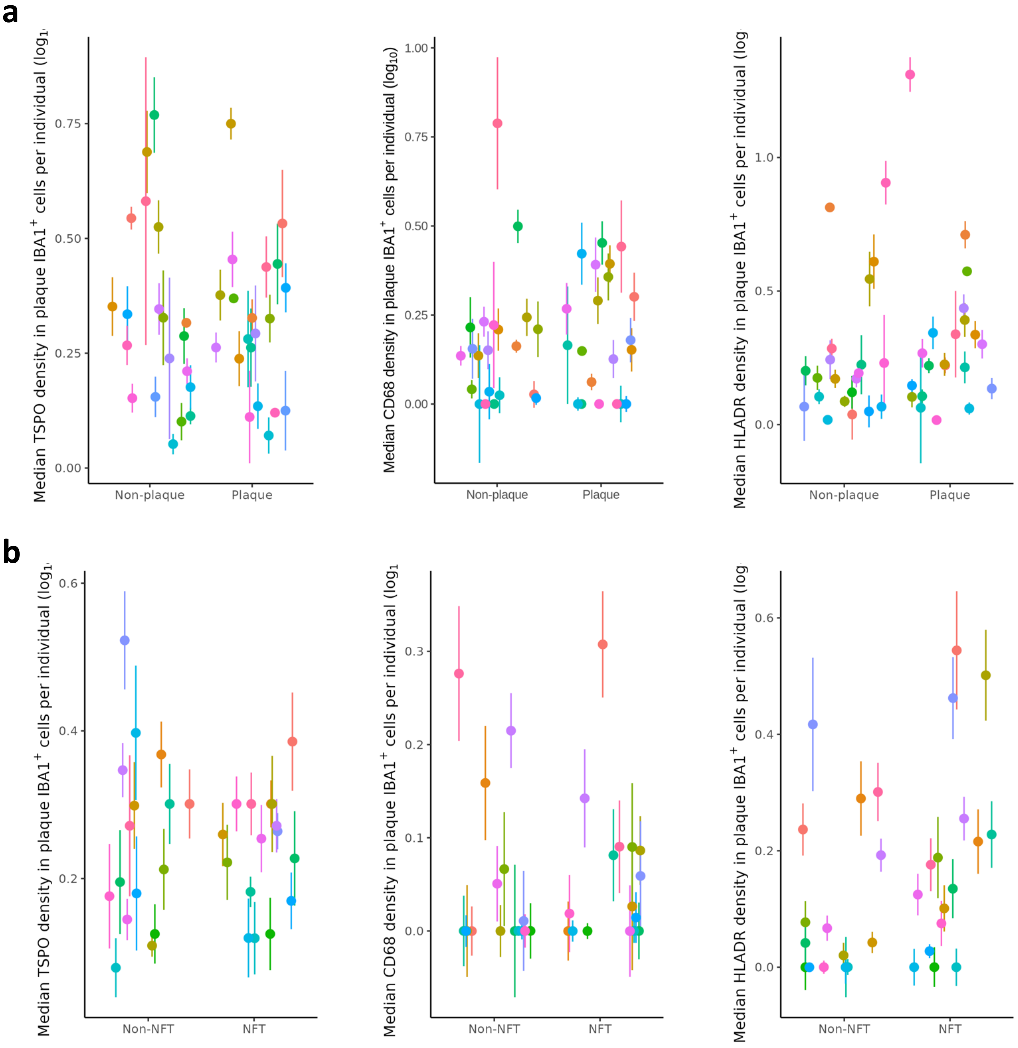

Figure S9. Correlation of TSPO with CD68 and HLA-DR in IBA1+ cells quantified by IMC in the AD brain. TSPO density shows no correlation with HLA-DR density and a significant but weak correlation with CD68 density in IBA1+ cells (Pearson's r^2=0.06). Source data are provided as a Source Data file.

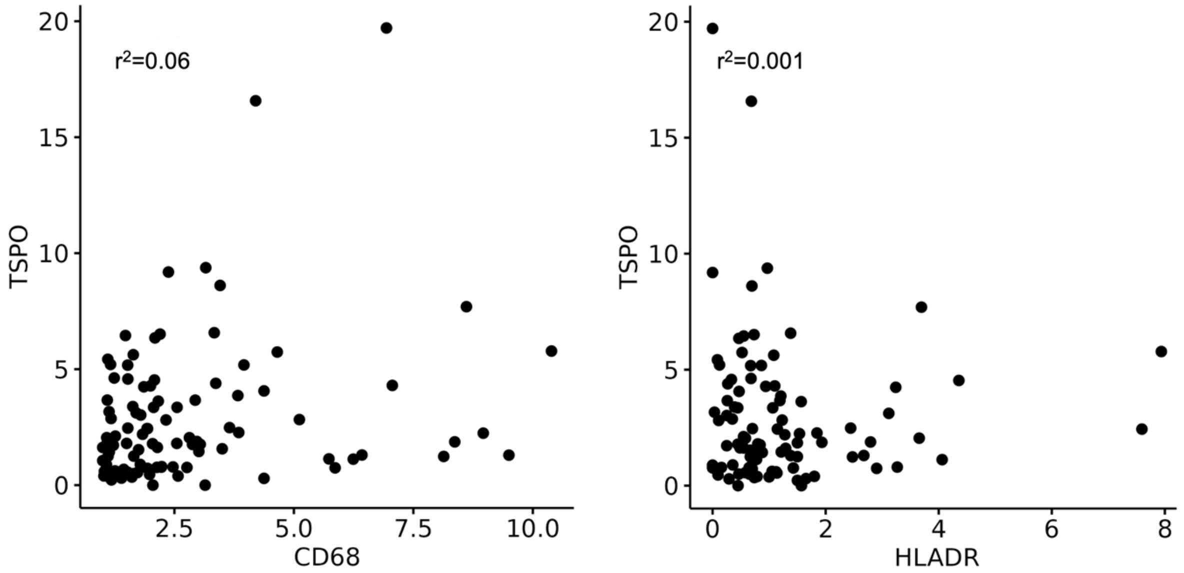

Figure S10. Identification of proinflammatory activated microglial subsets in human single cell RNA sequencing and single nucleus RNA sequencing datasets. **a** A subset of microglial cells shows a significant enrichment of proinflammatory (P=1.04E-87) and disease-associated gene sets (P=0) and a downregulation of a homeostatic gene set (P=1.64E-49) from a human single cell RNA sequencing study (Olah et al, upper row). Similarly, **b** the proinflammatory (P=1.42E-8), the disease-associated (P=1.72E-212) gene sets are significantly upregulated whereas the homeostatic gene set (P=1.13E-123) is significantly downregulated in a subset of microglia from a human single nucleus RNA sequencing study (Smith et al, lower row). Source data are provided as a Source Data file.

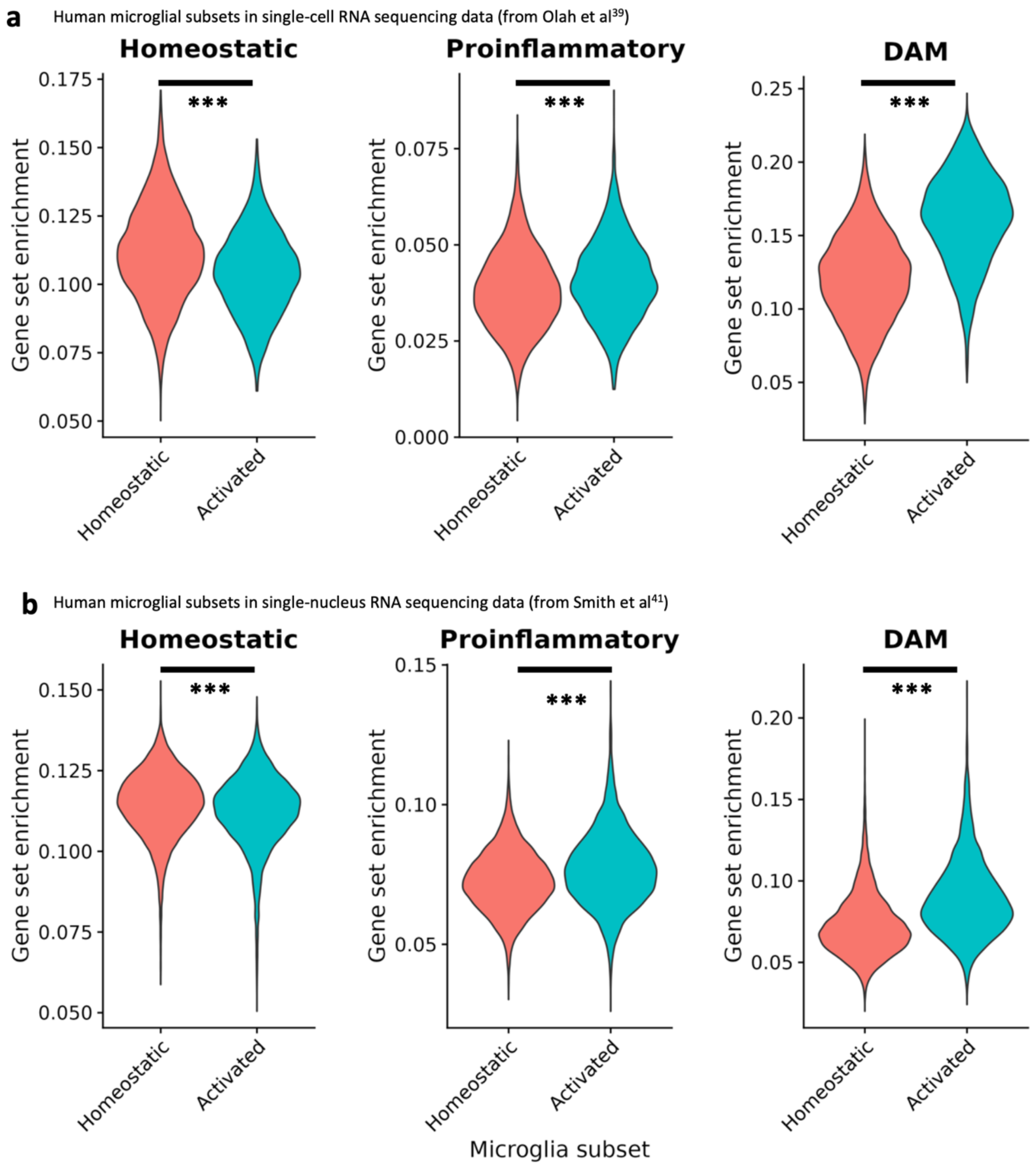

Figure S11. Violin plot of the expression of *TSPO* in vascular nuclei isolated from postmortem human brain (somatosensory and entorhinal cortex) samples from individuals with AD and non-demented controls (CON) showing an increase in *TSPO* expression in AD (logFC= 1.499, P=0.007, statistics table). 24 independent biological samples were used (n=12 NDC and 12 AD). For demonstration purposes, the *TSPO* violin plot only contains the nuclei where *TSPO* is expressed, although the statistical analysis was performed on pseudobulk data, thus on data from all nuclei. Source data are provided as a Source Data file.

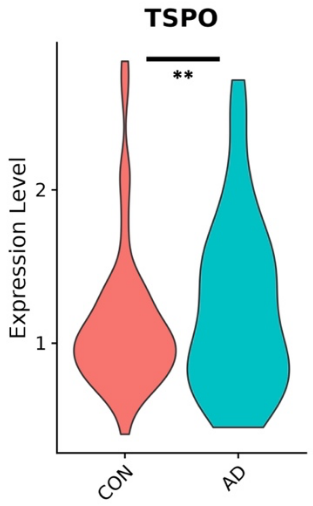

Figure S12. Tspo is significantly upregulated (P=12.51E-5) in proinflammatory activated subsets of microglia in a mouse single cell RNA sequencing dataset. Homeostatic P2ry12 is significantly downregulated (P=4.30E-79) whereas Apoe (P=6.24E-245), Trem2 (P=3.54E-15), Tyrobp (P=3.82E-74) and Cst7 (P=3.76E-155) are significantly upregulated, indicating proinflammatory activation (statistics table). The bulk of the TSPO expression values are higher in the DAM compared to the non-DAM cells. In scRNAseq, the expression value and the statistical analysis consider the number of cells that express the gene of interest over the total number of cells in each group. So, TSPO is expressed in proportionally more cells of the DAM compared to the non-DAM group. The statistical analysis, which thus considers the average expression value of TSPO in the two groups of microglia and the percentage of cells in each group that expressed TSPO at all, clearly confirms that TSPO is upregulated in DAM cells. Source data are provided as a Source Data file.

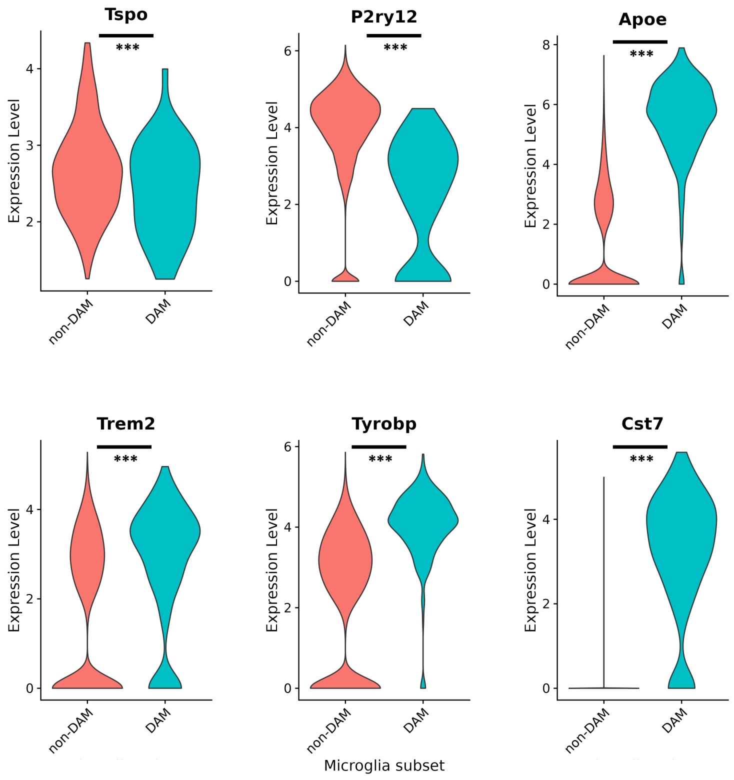

Figure S13. a Representative images of an acute lesion and control tissue in marmoset EAE (scale bar = 50 µm). **b,c** IBA1+ and TSPO+IBA1+ cells are increased in acute and subacute lesions compared to white matter in control marmoset. **d** TSPO expression in individual microglia, here defined as the percentage of TSPO+ pixels using immunofluorescence, was not increased in acute or subacute lesions relative to control (n = the number of cells per independent lesion analysed). Biologically independent samples were used in all experiments (n=1 CON and n=3 EAE). Bar graphs mark the mean ± SEM. Box and whiskers mark the 25th to 75th percentiles and min to max values, respectively, with the median indicated. Source data are provided as a Source Data file.

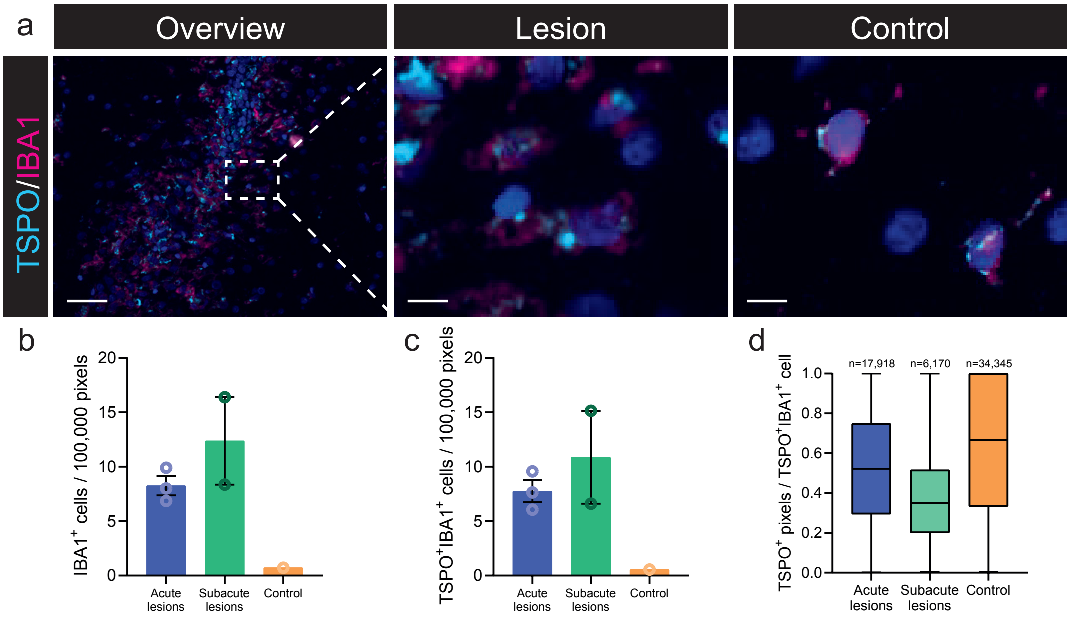

Figure S14. Planned and measured concentrations of XBD173 in medium for experiments described in Figures 8d and 8e. Source data are provided as a Source Data file.

Planned Concentration (nM)	Measured Concentration (nM)
XDB173	XDB173
Cell supernatant 1.78nM XBD	2.0
Cell supernatant 1.78nM XBD	1.9
Cell supernatant 22.5nM XBD	21.8
Cell supernatant 22.5nM XBD	20.6
Cell supernatant 284nM XBD	290.6
Cell supernatant 284nM XBD	318.8

Figure S15. Violin plots of the expression of *HK2, LCP2* and *TFEC* in brain cell types in a human AD snRNAseq dataset (Olig=oligodendrocytes, EN=excitatory neurons, IN= inhibitory neurons, OPC= oligodendrocyte precursor cells, Astro=astrocytes, Micro=microglia, Vasc= vascular cells). 88 independent biological samples were employed (statistics table). Pairwise comparisons of the expression of each one of these genes in microglia vs each one of the other cell clusters showed that all three genes are significantly more highly expressed in microglia compared to other cell types ($p<10^{-5}$ for all the comparisons). The comparison of microglial *LCP2* and *TFEC* expression to the expression in the other cell types showed higher logFC values (all between 0.58 and 0.87) than the same comparisons for *HK2* (logFC values between 0.37 and 0.43), indicating that *LCP2* and *TFEC* show a relatively more specific expression in microglia than *HK2.* Source data are provided as a Source Data file.

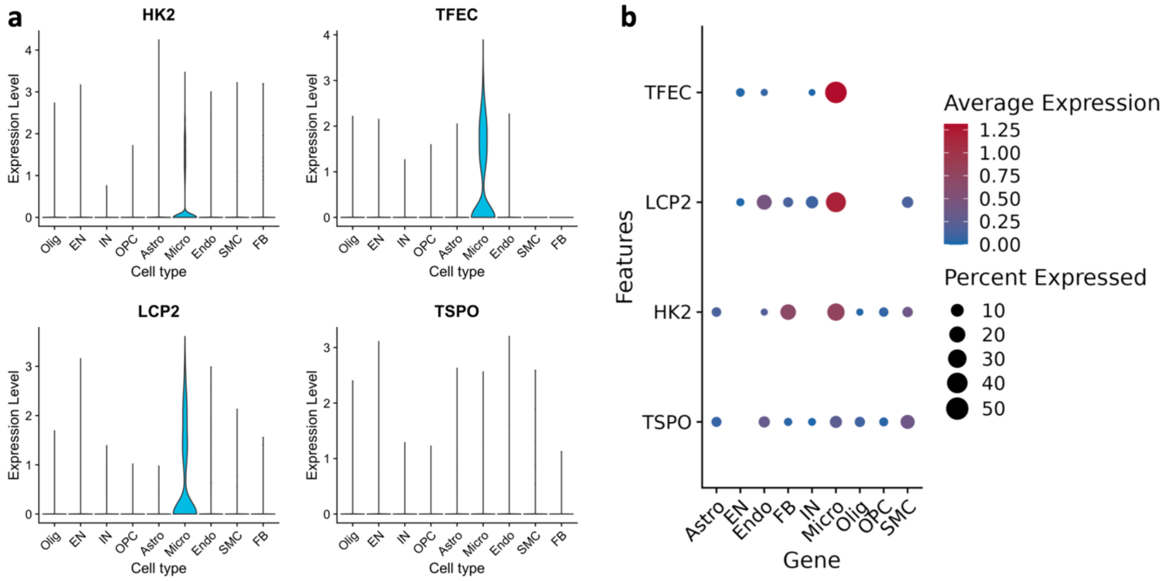

Figure S16 Effect of 24 hours stimulation with LPS (100ng/mL), IFNγ (10ng/mL) or IFNα (10ng/mL) of primary human macrophages *in vitro* on TFEC and LCP2 gene expression. Biologically independent samples were used for all experiments (n=4 for all conditions). Bar graphs mark the mean ± SEM. Source data are provided as a Source Data file.

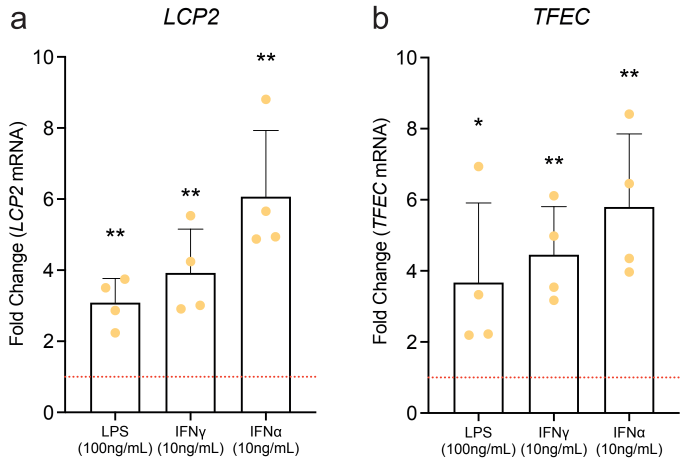

Figure S17 a Representative LCP2 IHC image in an AD and a NDC brain section (scale bar=120 μm). **b** The % LCP2 positive area in the hippocampus of AD samples is significantly higher than NDC samples (P= 0.00326). **c** Representative double immunofluorescence LCP2 and IBA1 staining images in MS brain sections (scale bar=25 μm for the active MS lesion and 10 μm for the NAWM) d The % double LCP2 and IBA1 positive area in active MS lesions is significantly higher than in NAWM (p=1.65E-06). **e** Representative double immunofluorescence LCP2 and IBA1 staining images in ALS and NDC spinal cord sections (Scale bar 120 μm) **f** The % double LCP2 and IBA1 positive area in the ALS is significantly higher than NDC samples (p=0.0034). Biologically independent samples were used for all experiments (**b** n=6 CON and n=6 AD)(**d** n=5 independent samples with 2 regions of interest)(**f** n=4 ALS and n=6 CON). Statistical significance was determined in **b** and **d** using a one-way ANOVA while using a mixed effects model for **f**. Box and whiskers mark the 95% confidence interval, respectively, with the median indicated. Source data are provided as a Source Data file.

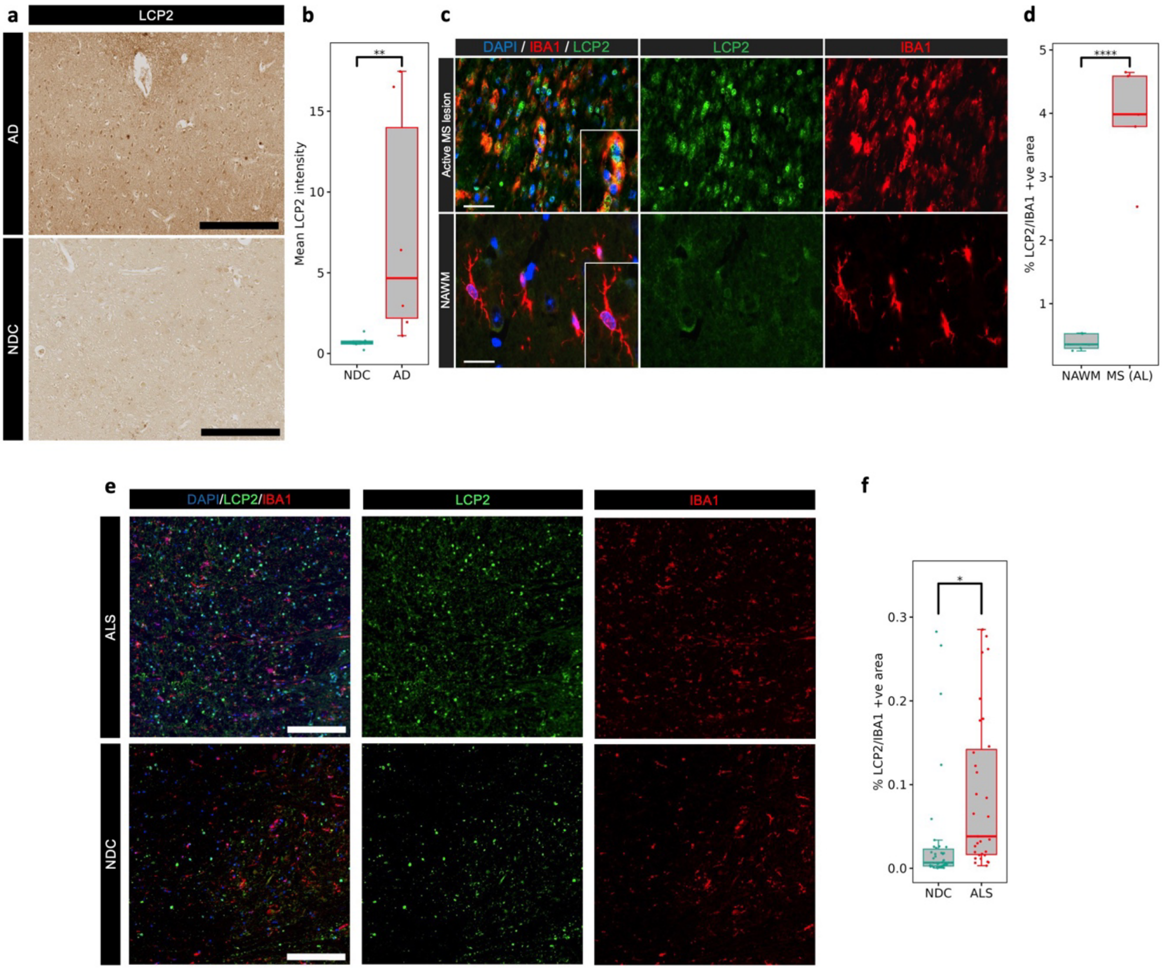

Figure S18. LCP2 Antibody controls for **a** AD IHC experiment (scale bar=100µm) **b** MS IF experiment (scale bar=40 µm) **c** For the ALS experiment cohort (scale bar=100 µm) a different secondary antibody for the LCP2 staining from the one of the MS cohort was used. All the other antibodies and the conditions of this experiment were the same. This figure shows that the secondary antibody for the LCP2 staining does not bind the primary or secondary antibodies used for the IBA1 staining. Source data are provided as a Source Data file.

a

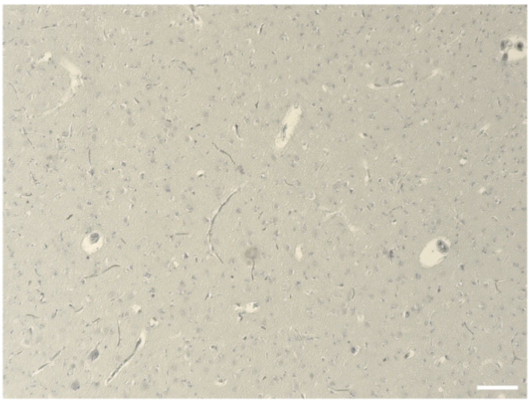

b

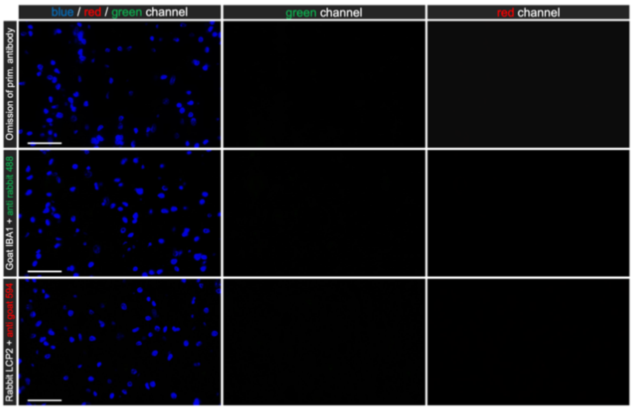

c

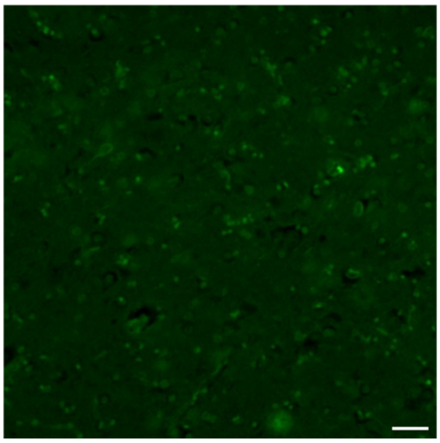

Figure S19. Schematic overview of mouse models and strains, the timepoints of age at which the mice were studied, the areas that were sampled and how they were sampled. Source data are provided as a Source Data file.

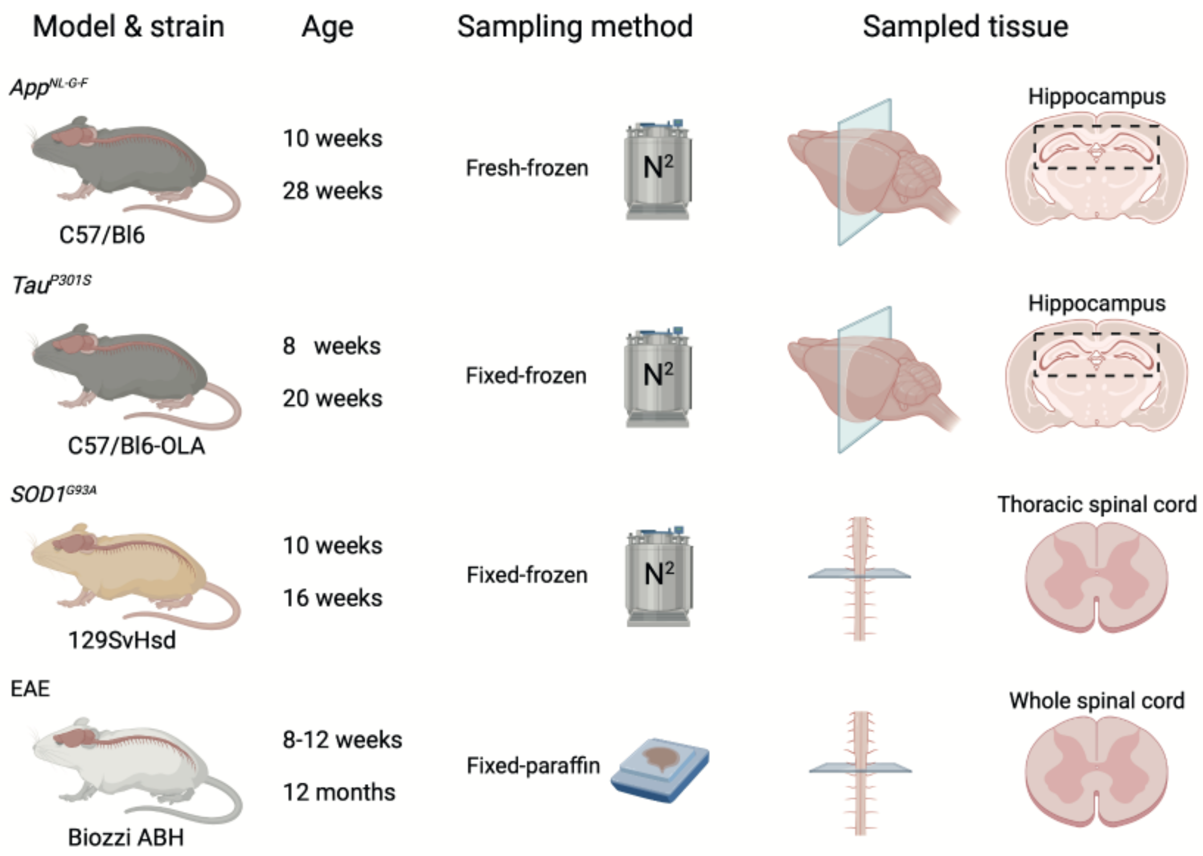

Figure S20: TSPO+ area quantification. Images were taken of microglia or astrocytes and individual cells were outlined using the drawing tools in ImageJ, masks were saved for later reference. Fluorescent channels were separated after which the TSPO channel was used for analysis, masks were then re-applied. Next, a threshold was used within the masks for TSPO+ signal and quantified and converted to TSPO+ area (um^2) for each individual cell and used for analysis. Source data are provided as a Source Data file.

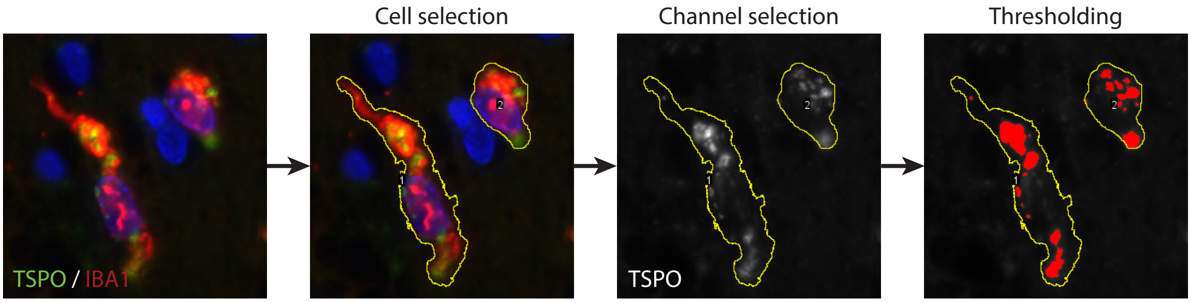

Figure S21 Violin plot of the expression of TSPO in microglial nuclei isolated from postmortem human brain (cingulate cortex) samples from individuals with bipolar disorder (BD, n=5) and non-neurological/non-psychiatric controls (CON, n=7). For demonstration purposes, the TSPO violin plot only contains the nuclei where TSPO is expressed, although the statistical analysis was performed on pseudobulk data, thus on data from all nuclei. Source data are provided as a Source Data file.

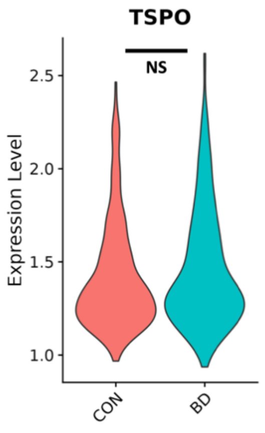

Supplementary Table 1. Clinical details of AD and control cases for IF cohort

Case	Diagnosis	Region	Braak stage
AD cases			
1	AD	HC CA4	6
2	AD	HC CA4	6
3	AD	HC CA4	6
4	AD	HC CA4	6
5	AD	HC CA4	6
Controls			
1	NDC	HC CA4	2
2	NDC	HC CA4	3
3	NDC	HC CA4	3
4	NDC	HC CA4	2
5	Ischemic changes	HC CA4	4

Abbreviations: HC – hippocampus; NDC – non-demented control; PMD – postmortem delay. Age in years (Average±SD): AD cases: 74.2±11.09, Controls: 78.8±9. Sex: AD cases: 3 females, 2 males, Controls: 4 females, 1 male. Postmortem delay (Average±SD in minutes): AD: 350±46, Controls: 350±58.

Supplementary Table 2. Clinical details of AD and control cases for Confocal cohort

Case	Diagnosis	Region	Braak stage	Rs6971 Genotype
AD cases				
1	AD	CA4	5	1
2	AD	CA4	5	2
3	AD	CA4	5	3
4	AD	CA4	5	4
5	AD	CA4	5	5
6	AD	CA4	5	6
Controls				
1	NDC	CA4	0	1
2	NDC	CA4	2	2
3	NDC	CA4	2	3
4	NDC	CA4	2	4
5	NDC	CA4	3	5
6	NDC	CA4	3	6

Abbreviations: PMD – postmortem delay; CA4 – CA4 region of the hippocampus. Age in years (Average±SD): AD cases: 82±7, Controls: 79±5. Sex: AD cases: 3 females, 3 males, Controls: 3 females, 3 males. Postmortem delay (Average±SD in hours): AD: 52±19, Controls: 44±22.

Supplementary Table 3. Clinical details of AD and control cases for [^{125}I]CLINDE autoradiography and western blotting cohort.

Case	Diagnosis	Region	Braak stage	Rs6971 Genotype
AD cases				
1	AD	HC	6	CC
2	AD	HC	5	CC
3	AD	HC	5	CC
4	AD	HC	5	CT
5	AD	HC	6	CT
6*	AD	HC	5	CC
7*	AD	HC	5	CC
8*	AD	HC	5	CC
Controls				
1	NDC	HC	0	CT
2	NDC	HC	1	CC
3	NDC	HC	2	CC
4	NDC	HC	2	CC
5	NDC	HC	2	CT
6	NDC	HC	2	CC
7*	NDC	HC	2	CT
8*	NDC	HC	2	CT
9^	NDC	HC	2	Unknown
10^	NDC	HC	2	Unknown

Abbreviations: HC – hippocampus; NDC – non-demented control; PMD – postmortem delay. Age in years (Average±SD): AD cases: 91.8±6, Controls: 86.3±5. Sex: AD cases: 4 females, 4 males, Controls: 5 females, 5 males. Postmortem delay (Average±SD in hours): AD: 7±5, Controls: 11±11.

*Autoradiography only as insufficient tissue for western

^Western blot only as genotype unknown and therefore autoradiography signal not interpretable

Supplementary Table 4. Clinical details of AD cases for IMC cohort

Case	Diagnosis	TSPO genotype	Region	Braak stage
1	AD	CT	EC, MTG	5
2	AD	CT	EC, MTG	5
3	AD	CC	EC, MTG	5
4	AD	CT	EC, MTG	5
5	AD	CC	EC, MTG	6
6	AD	TT	EC, MTG	6
7	AD	CC	EC, MTG	6
8	AD	CC	EC, MTG	5
9	AD	CT	EC, MTG	6
10	AD	CT	EC, MTG	6
11	AD	NA	EC, MTG	3
12	AD	TT	EC, MTG	5
13	AD	TT	EC, MTG	6
14	AD	CT	EC, MTG	5
15	AD	CT	EC, MTG	3
16	AD	CT	EC, MTG	5
17	AD	CC	EC, MTG	6
18	AD	CT	EC, MTG	5
19	AD	CT	EC, MTG	5
20	AD	CT	EC, MTG	5
21	AD	NC	EC, MTG	6
22	AD	CC	EC, MTG	6

Abbreviations: EC – entorhinal cortex; MTG – medial temporal gyrus; PMD – postmortem delay. Age in years (Average±SD): 73±10
Sex: 11 females, 11 males. Postmortem delay (Average±SD in hours): 24±14.

Supplementary Table 5. Clinical details of ALS and control cases

Case	Diagnosis	DD, months	Cause of death	SPC levels
ALS short disease duration				
1	sALS	6	respiratory failure	C/T/L
2	sALS	7	respiratory failure	C/T/L
3	sALS	12	euthanasia	C/T/L
4	sALS	12	euthanasia	C/T/L
5	sALS	12	respiratory failure	T/L
6	sALS	13	euthanasia	C/T
7	sALS	16	euthanasia	C/T/L
ALS medium disease duration				
8	sALS	36	unknown	C/T/L
9	fALS	57	pneumonia	C/T/L
10	sALS	87	euthanasia	C/T/L
11	sALS (C9orf72)	107	pneumonia	C/T/L
Controls				
12	bricker-bladder	N/A	lung embolism	C/T/L
13	kidney carcinoma	N/A	lung embolism	C/T/L
14	heart ischemia	N/A	endocarditis	C/T/L
15	adenocarcinoma	N/A	paralytic ileus	C/T/L
16	oesophagus carcinoma	N/A	multi-organ failure	C/T/L
17	cholangio-carcinoma	N/A	multi-organ failure	T
18	COPD, pneumonia	N/A	respiratory failure	C/T
19	pleuritis carcinomatosis	N/A	respiratory failure	C/T/L
20	pancreas carcinoma	N/A	abdominal bleeding	C/T/L
21	gallbladder carcinoma	N/A	heart failure	C/T/L

Abbreviations: C – cervical; COPD – chronic obstructive pulmonary disease; DD – disease duration; fALS – familial ALS; L – lumbar; PMD – postmortem delay; S – sacral; sALS – sporadic ALS; SPC – spinal cord; T – thoracic. Age in years (Average±SD): ALS short disease duration cases: 67.8±11, ALS medium disease duration cases: 63.5±15, Controls: 64.9±11. Sex: ALS short disease duration cases: 4 females, 3 males, ALS medium disease duration cases: 1 female, 3 males, Controls: 6 females, 4 males. Postmortem delay was less than 12 hours for ALS cases and less than 48 hours for controls. Primary onset in ALS cases was either in the legs (n=4), arms (n=5), respiratory (n=1), or bulbar (n=1).

.

Supplementary Table 6. Clinical details of MS and control cases

Case	Diagnosis	Disease duration, years	Cause of death
MS cases			
1	SPMS	10	Euthanasia
2	SPMS	27	Respiratory failure
3	SPMS	18	Euthanasia
4	SPMS	21	Unknown
5	Unknown	Unknown	Unknown
MS cases for LPC2 validation			
1	SPMS	Unknown	Infection
2	SPMS	10	Euthanasia
3	PPMS	24	Pulmonary insufficiency
4	PPMS	32	Euthanasia
5	SPMS	21	Infection
Controls			
1	NNC	N/A	Heart failure
2	NNC	N/A	Pneumonia
3	NNC	N/A	Heart failure
4	NNC	N/A	Mamma carcinoma
5	NNC	N/A	Pneumonia

Abbreviations: N/A – not applicable; NNC – non-neurological control; SPMS – secondary progressive multiple sclerosis. Age in years (Average±SD): MS cases: 53.5±10, Controls: 82.4±6. Sex: MS cases: 7 females, 3 males, Controls: 3 females, 2 males. Postmortem delay (Average±SD in minutes): MS cases: 606±49, MS cases for LPC2: 649±67, Controls: 366±89.

Supplementary Table 7. Clinical History of mice with EAE

Mouse number	Sampling day	Age (weeks)
Acute young (aEAE)		
1	14 (4)	10-15
2	12 (4)	10-15
3	15 (4.5)	10-15
4	15 (4)	10-15
5	13 (4.5)	10-15
6	20 (4.5)	10-15
Acute old (PEAE)		
1	15 (4.5)	> 50
2	13 (5)	> 50
3	13 (4.5)	> 50
4	16 (4.5)	> 50
5	15 (5)	> 50
6	17 (4.5)	> 50

EAE mice were immunized with SCH in CFA and monitored (sampling day refers to the day after immunization). Indicated clinical scores are the maximal scores during neurological episodes of EAE. Abbreviations: EAE – experimental autoimmune encephalomyelitis; aEAE – acute EAE, PEAE – progressive EAE.

Supplementary Table 8. Clinical History of Marmosets

Animal ID	Gender	Disease Status	Age at EAE induction (years)	Disease duration (days)	Age (years)
1	M	Control	N/A	N/A	3
4	F	EAE	2.0	32	2.1
5	F	EAE	1.6	105	1.9
8	F	EAE	1.6	123	1.9

Abbreviations: EAE – experimental autoimmune encephalitis; N/A – not applicable.

Supplementary Table 9. Antibodies for immunofluorescence, and imaging mass cytometry

Antigen	Species (isotype)	Clonality	Dilution	Ln-Isotope	Antigen Retrieval	Product Number	Supplier
TSPO	goat	pAb	1:750	NA	Citrate	NB100-41398	Novus Biologicals
TSPO	rabbit	mAb	1:750	NA	Citrate	AB109497	Abcam
IBA1	rabbit	pAb	1:10000	NA	Tris-EDTA	019-19741	Wako
IBA1	goat	pAb	1:1000	NA	Tris-EDTA	AB48004	Abcam
IBA1	guinea pig	pAb	1:100	NA	Citrate	234004	Synaptic Systems
GFAP	chicken	pAb	1:500	NA	Citrate	AB5541	Millipore
HLA-DR	mouse (IgG2B)	mAb	1:750	NA	Citrate	14-9956-82	Invitrogen
Aβ IC16	mouse (IgG2A)	mAb	1:400	NA	Citrate	N/A	in-house[a]
MX04	NA	NA	1:1000	NA	NA	4950	Tocris
P-Tau AT8	mouse (IgG1)	mAb	1:400	NA	Citrate	AB_223647	Invitrogen
P-TAU AT8	Mouse	mAb	1:400	165Ho	EDTA	MN1020	ThermoFisher
PLP	mouse (IgG2A)	mAb	1:200	NA	Citrate	MCA839G	Bio-Rad
LCP2	rabbit	pAB	1:50	NA	Tris-EDTA	Ab196599	Abcam
LCP2	rabbit	pAB	1:100	NA	Tris-EDTA	100728	LSBiosciences
CD68 (IMC)	mouse	mAb	1:800	159Tb	EDTA	3159035D	Fluidigm
GFAP (IMC)	rabbit	mAb	1:600	162Dy	EDTA	Ab218309	Abcam
HLA-DR (IMC)	mouse	mAb	1:400	174Yb	EDTA	3174025D	Fluidigm
IBA1 (IMC)	rabbit	pAb	1:3000	169Tm	EDTA	019-197471	Wako
TSPO (IMC)	rabbit	mAb	1:400	149Sm	EDTA	Ab213654	Abcam
Aβ 4G8 (IMC)	Mouse	mAb	1:500	144nD	EDTA	800702	Biolegend
P-TAU AT8 (IMC)	Mouse	mAb	1:400	165Ho	EDTA	MN1020	ThermoFisher

[a]With permission from Carsten Korth, Heinrich Heine University, Düsseldorf, Germany. Abbreviations: GFAP – glial fibrillary acidic protein; IBA1 – ionized calcium-binding adaptor molecule 1; mAb – monoclonal antibody; pAb – polyclonal antibody; P-Tau – phosphorylated Tau (Ser202, Thr205).

Supplementary Table 10. Human Genes with the AP1 binding site in the core promoter region which are upregulated in human myeloid cells after exposure to a proinflammatory stimulus *in vitro* (See Fig. 1b for in vitro experiments)

ACOX3	CEBPB	DUSP5	JAG1	NADK	PPP1R15A	SLC7A11	TNFAIP6
ADM	CFLAR	EIF5B	JUNB	NFKBIA	PPP2CA	SMARCA5	TNFSF9
ARIH1	CHST7	EXT1	KDM6A	NINJ1	PTGER4	SNN	TP53BP2
B4GALT5	CLIC4	GCLM	KLF7	NOCT	PTS	STAT5A	UBE2Z
BCL6	CSRNP2	GNA15	KLF9	OAS3	RELB	STK26	UBXN1
BHLHE40	CTNNBL1	GPR183	LCP2	OLR1	RERE	STX12	USP25
BST2	CYB5A	GTF2B	LRP10	PANX1	RHOG	SUSD6	VCAN
BTG1	CYP20A1	HBEGF	LRP4	PARP12	RIPK2	TANK	VIM
CD48	DDX21	HK2	MAP3K5	PELI1	RMC1	TFEC	WTAP
CD80	DDX56	IFIH1	MARCKSL1	PLEK	RTCB	TGM2	ZFP36
CDC42EP4	DNTTIP2	IL18	MCL1	PLGRKT	SDC4	TIMP1	ZNF277
CDK12	DRAM1	IRF2	MYO10	PPP1R12A	SERPINB9	TMEM140	ZNF823

Supplementary Table 11. Clinical details of bipolar disorder and control cases

Case	Diagnosis	Region	PMD, h:min
BD cases			
1	BD	Cingulate cortex	06:20
2	BD	Cingulate cortex	04:40
3	BD	Cingulate cortex	10:15
4	BD	Cingulate cortex	09:15
5	BD	Cingulate cortex	06:00
6	BD	Cingulate cortex	05:45
7	BD	Cingulate cortex	07:35
Controls			
1	CON	Cingulate cortex	05:05
2	CON	Cingulate cortex	07:30
3	CON	Cingulate cortex	08:30
4	CON	Cingulate cortex	04:50
5	CON	Cingulate cortex	04:20

Abbreviations: BD – bipolar disorder; CON – Control; PMD – postmortem delay; Age in years (Average±SD): BD cases: 75.3±11, Controls: 79±16. Sex: BD cases: 5 females, 2 males, Controls: 3 females, 2 males.

Supplementary Table 12. Public datasets used in this study.

Fig.	Citation	Dataset Link
1a	107-110	https://www.ebi.ac.uk/biostudies/arrayexpress/studies/E-GEOD-35825?query=E-GEOD-35825<br>https://www.ebi.ac.uk/biostudies/arrayexpress/studies/E-GEOD-53986?query=E-GEOD-53986<br>https://www.ebi.ac.uk/biostudies/arrayexpress/studies/E-GEOD-14769?query=E-GEOD-14769<br>https://www.ebi.ac.uk/biostudies/arrayexpress/studies/E-GEOD-15610?query=E-GEOD-15610<br>https://www.ebi.ac.uk/biostudies/arrayexpress/studies/E-GEOD-8621?query=E-GEOD-8621<br>https://www.ebi.ac.uk/biostudies/arrayexpress/studies/E-GEOD-69607?query=E-GEOD-69607<br>https://www.ebi.ac.uk/biostudies/arrayexpress/studies/E-GEOD-72518?query=E-GEOD-72518
1b	111-120	https://www.ebi.ac.uk/biostudies/arrayexpress/studies/E-GEOD-32282?query=E-GEOD-32282<br>https://www.ebi.ac.uk/biostudies/arrayexpress/studies/E-GEOD-18816?query=E-GEOD-18816<br>https://www.ebi.ac.uk/biostudies/arrayexpress/studies/E-GEOD-16755?query=E-GEOD-16755<br>https://www.ebi.ac.uk/biostudies/arrayexpress/studies/E-GEOD-79077?query=E-GEOD-79077<br>https://www.ebi.ac.uk/biostudies/arrayexpress/studies/E-MTAB-5913?query=E-MTAB-5913<br>https://www.ebi.ac.uk/biostudies/arrayexpress/studies/E-MTAB-5917?query=E-MTAB-5917<br>https://www.ebi.ac.uk/biostudies/arrayexpress/studies/E-GEOD-1432?query=E-GEOD-1432<br>https://www.ebi.ac.uk/biostudies/arrayexpress/studies/E-GEOD-8515?query=E-GEOD-8515<br>https://www.ebi.ac.uk/biostudies/arrayexpress/studies/E-GEOD-61535?query=E-GEOD-61535<br>https://www.ebi.ac.uk/biostudies/arrayexpress/studies/E-GEOD-19315?query=E-GEOD-19315<br>https://www.ebi.ac.uk/biostudies/arrayexpress/studies/E-GEOD-19482?query=E-GEOD-19482<br>https://www.ebi.ac.uk/biostudies/arrayexpress/studies/E-GEOD-32141?query=E-GEOD-32141<br>https://www.ebi.ac.uk/biostudies/arrayexpress/studies/E-GEOD-36933?query=E-GEOD-36933<br>https://www.ebi.ac.uk/biostudies/arrayexpress/studies/E-GEOD-41295?query=E-GEOD-41295<br>https://www.ebi.ac.uk/biostudies/arrayexpress/studies/E-GEOD-43596?query=E-GEOD-43596<br>https://www.ebi.ac.uk/biostudies/arrayexpress/studies/E-GEOD-5099?query=E-GEOD-5099<br>https://www.ebi.ac.uk/biostudies/arrayexpress/studies/E-GEOD-24897?query=E-GEOD-24897<br>https://www.ebi.ac.uk/biostudies/arrayexpress/studies/E-GEOD-13670?query=E-GEOD-13670
1h	30	https://www.ncbi.nlm.nih.gov/geo/query/acc.cgi?acc=GSE38377
1i	29	https://www.ncbi.nlm.nih.gov/geo/query/acc.cgi?acc=GSE66594
1g, S2c	This study	https://www.ncbi.nlm.nih.gov/geo/query/acc.cgi?acc=GSE236999
2d	50,108,118,121-123	https://www.ebi.ac.uk/biostudies/arrayexpress/studies/E-GEOD-19315?query=E-GEOD-19315<br>https://www.ebi.ac.uk/biostudies/arrayexpress/studies/E-MTAB-7572?query=E-MTAB-7572<br>https://www.ebi.ac.uk/biostudies/arrayexpress/studies/E-GEOD-15610?query=E-GEOD-15610<br>https://www.ebi.ac.uk/biostudies/arrayexpress/studies/E-GEOD-53986?query=E-GEOD-53986<br>https://www.ebi.ac.uk/biostudies/arrayexpress/studies/E-MEXP-3469?query=E-MEXP-3469<br>https://www.ebi.ac.uk/biostudies/arrayexpress/studies/E-GEOD-6353?query=E-GEOD-6353<br>https://www.ebi.ac.uk/biostudies/arrayexpress/studies/E-GEOD-59263?query=E-GEOD-59263<br>https://www.ncbi.nlm.nih.gov/geo/query/acc.cgi?acc=GSE71037<br>https://www.ebi.ac.uk/biostudies/arrayexpress/studies/E-GEOD-30956?query=E-GEOD-30956
4e, S10a	39	https://www.synapse.org/#!Synapse:syn21438358
4f, S10b, S11	41	https://www.ncbi.nlm.nih.gov/geo/query/acc.cgi?acc=GSE160936
8j-l	This study<br>50-53,55,57,124	https://www.ncbi.nlm.nih.gov/geo/query/acc.cgi?acc=GSE100382<br>https://www.ncbi.nlm.nih.gov/geo/query/acc.cgi?acc=GSE55536<br>https://www.ebi.ac.uk/biostudies/arrayexpress/studies/E-MTAB-7572?query=E-MTAB-7572<br>https://www.ncbi.nlm.nih.gov/geo/query/acc.cgi?acc=GSE57494<br>https://www.ncbi.nlm.nih.gov/geo/query/acc.cgi?acc=GSE236998<br>https://www.ncbi.nlm.nih.gov/geo/query/acc.cgi?acc=GSE103958<br>https://www.ncbi.nlm.nih.gov/geo/query/acc.cgi?acc=GSE62641<br>https://www.ncbi.nlm.nih.gov/geo/query/acc.cgi?acc=GSE82043<br>https://www.ncbi.nlm.nih.gov/geo/query/acc.cgi?acc=GSE58318<br>https://www.ebi.ac.uk/biostudies/arrayexpress/studies/E-ERAD-165?query=E-ERAD-165%20
S3a, S3b	30,125	https://www.ncbi.nlm.nih.gov/geo/query/acc.cgi?acc=GSE36952<br>https://www.ncbi.nlm.nih.gov/geo/query/acc.cgi?acc=GSE38371
S12	40	https://www.ncbi.nlm.nih.gov/geo/query/acc.cgi?acc=GSE98969
