# Supplementary material for: Translocator protein is a marker of activated microglia in rodent models but not human neurodegenerative diseases
Source: Nat Commun. 2023 Aug 28;14:5247. doi: 10.1038/s41467-023-40937-z (PMC10462763; doi:10.1038/s41467-023-40937-z)
Supplement: Supplementary file 3 — Description of Additional Supplementary Files [file 41467_2023_40937_MOESM3_ESM.pdf]

Description of Additional Supplementary Files

File name: Supplementary Data 1

Description: Human and mouse WGCNA TSPO modules and GO analyses

File name: Supplementary Data 2

Description: Multiple sequence alignment and phylogenetic tree construction

File name: Supplementary Data 3

Description: Statistical file
