# Supplementary material for: Translocator protein is a marker of activated microglia in rodent models but not human neurodegenerative diseases
Source: Nat Commun. 2023 Aug 28;14:5247. doi: 10.1038/s41467-023-40937-z (PMC10462763; doi:10.1038/s41467-023-40937-z)
Supplement: Supplementary file 7 — Reporting Summary [file 41467_2023_40937_MOESM7_ESM.pdf]

nature portfolio

Corresponding author(s): David R. Owen

Last updated by author(s): Jul 23, 2023

Reporting Summary

Nature Portfolio wishes to improve the reproducibility of the work that we publish. This form provides structure for consistency and transparency in reporting. For further information on Nature Portfolio policies, see our Editorial Policies and the Editorial Policy Checklist.

Statistics

For all statistical analyses, confirm that the following items are present in the figure legend, table legend, main text, or Methods section.

n/a	Confirmed	
☐	☒	The exact sample size (*n*) for each experimental group/condition, given as a discrete number and unit of measurement
☐	☒	A statement on whether measurements were taken from distinct samples or whether the same sample was measured repeatedly
☐	☒	The statistical test(s) used AND whether they are one- or two-sided<br>*Only common tests should be described solely by name; describe more complex techniques in the Methods section.*
☐	☒	A description of all covariates tested
☐	☒	A description of any assumptions or corrections, such as tests of normality and adjustment for multiple comparisons
☐	☒	A full description of the statistical parameters including central tendency (e.g. means) or other basic estimates (e.g. regression coefficient) AND variation (e.g. standard deviation) or associated estimates of uncertainty (e.g. confidence intervals)
☐	☒	For null hypothesis testing, the test statistic (e.g. *F*, *t*, *r*) with confidence intervals, effect sizes, degrees of freedom and *P* value noted<br>*Give P values as exact values whenever suitable.*
☐	☒	For Bayesian analysis, information on the choice of priors and Markov chain Monte Carlo settings
☒	☐	For hierarchical and complex designs, identification of the appropriate level for tests and full reporting of outcomes
☐	☒	Estimates of effect sizes (e.g. Cohen's *d*, Pearson's *r*), indicating how they were calculated

Our web collection on statistics for biologists contains articles on many of the points above.

Software and code

Policy information about availability of computer code

Data collection	Public datasets used in this study were directly downloaded from "ArrayExpress", "Gene Expression Omnibus" or "Synapse" and no software was used. ImageJ (v1.53c) and Qupath (v0.4.2) were used for image quantification. SparkControl (Tecan) was used for absorbance measurements of ELISAs, Gen5 (BioTek), and CellQuest (BD Biosciences) were used for fluoresence acquisition of phagocytosis experiments. We have used SEA (Simple Enrichment Analysis) from the MEME-suite (v 5.4.1) to calculate the relative motif enrichment between Muroidea family species and non-Muroidea mammals. Pan-cellular and microglia masking was done using Ilastik (v1.1.3post3), CellProfiler (v4.2.1) and HistoCAT (v1.76)
Data analysis	R version 3.6.3 or 4.1.2 and Graphpad Prism 9.1 were used for data analysis.<br>R Packages: Limma v.3.42.2, meta v.5.1.1, Bowtie2 v.2.2.9, HOMER v.4.11.1, T-Coffee v13.45.0.4846264, Jalview v2.11.1.6, DESeq2 v.1.26.0, Seurat v3.0, nf-core/scflow, WGCNA v. 1.69, WebGestaltR v. 0.4.3, glmmTMB v10.32614/RJ-2017-066.

For manuscripts utilizing custom algorithms or software that are central to the research but not yet described in published literature, software must be made available to editors and reviewers. We strongly encourage code deposition in a community repository (e.g. GitHub). See the Nature Portfolio guidelines for submitting code & software for further information.

Data

Policy information about availability of data

All manuscripts must include a data availability statement. This statement should provide the following information, where applicable:

- Accession codes, unique identifiers, or web links for publicly available datasets
- A description of any restrictions on data availability
- For clinical datasets or third party data, please ensure that the statement adheres to our policy

The data that support the findings of this study are available in this manuscript and the Supplementary Information. Source data are provided with this paper.

Research involving human participants, their data, or biological material

Policy information about studies with human participants or human data. See also policy information about sex, gender (identity/presentation), and sexual orientation and race, ethnicity and racism.

Reporting on sex and gender	Both sexes were used in the study for human and animal subjects in both control and disease related groups. Sex is indicated in the supplementary information.
Reporting on race, ethnicity, or other socially relevant groupings	Race or ethnicity were not considered in this study as this information was not available for human postmortem tissue
Population characteristics	the study used multiple cohorts of post mortem tissues from non-neurological controls as well as people with MS, AD, and ALS. For details on population characteristics please see the supplementary tables describing age, sex, diagnosis, and cause of death for all subjects.
Recruitment	Tissue was obtained from multiple autopsy protocols where prospective informed consent was obtained for all subjects. For the samples used in the ALS cohort: Human tissue was obtained at autopsy at the department of Neuropathology of the Amsterdam UMC. For the samples used in the IF only cohorts (AD and MS): The rapid autopsy regimen of the Netherlands Brain Bank in Amsterdam (coordinator Prof I. Huitinga) was used to acquire the samples. For the AD samples used in the confocal and autoradiography cohort: The human brain samples were obtained from the Geneva Brain Bank. For the AD samples used in the IMC cohort: This study was carried out in accordance with the Regional Ethics Committee and Imperial College Use of Human Tissue guidelines. Cases were selected based first on clinical and neuropathological diagnosis from UK brain banks (London Neurodegeneration [King's College London], Newcastle Brain Tissue Resource, Queen's Square Brain Bank [UCL], Manchester Brain Bank, Oxford Brain Bank and Parkinson's UK [Imperial College London] Brain Bank).
Ethics oversight	All tissue was collected with the approval of the Medical Ethical Committee of the local hospitals and universities. All participants had given prospective informed consent for autopsy and use of their tissue for research purposes.

Note that full information on the approval of the study protocol must also be provided in the manuscript.

Field-specific reporting

Please select the one below that is the best fit for your research. If you are not sure, read the appropriate sections before making your selection.

☒ Life sciences ☐ Behavioural & social sciences ☐ Ecological, evolutionary & environmental sciences

For a reference copy of the document with all sections, see nature.com/documents/nr-reporting-summary-flat.pdf

Life sciences study design

All studies must disclose on these points even when the disclosure is negative.

Sample size	Sample sizes were dependent on availability of post-mortem material of neurological diseases and animal models. all sample sizes are stated in the statistical supplementary data and in the source data. For cell work, sample size was not predetermined and chosen based on expected heterogeneity: For human primary cells, samples size was chosen based on donor availability. The mouse primary cell sample size was matched to the human sample size. BV2 cell line: a minimum of 3 was chosen due to the high reproducibility of cell lines. For iPSC-microglialike cells, a minimum of two replicates from three differentiation batches was used to control for batch effects.
Data exclusions	no data was excluded from this study
Replication	For immunohistochemistry images were counted by multiple observers with a high correlation coefficient > 0.9. IHC data indicates a minimum of 3 biological repeats. For cell work, all attempts at replication were successful, data represents a minimum of 3 technical replicates.
Randomization	Randomisation was not applicable for human tissue assessment and mouse genotypes compared to wildtype. For immunohistochemistry images were taken randomly in regions of interest. For cell work all samples were treated equally.
Blinding	For immunohistochemistry and imaging mass cytometry images were blinded for analysis. For cell work, the investigators were not blinded during samples collection or analysis.

Reporting for specific materials, systems and methods

We require information from authors about some types of materials, experimental systems and methods used in many studies. Here, indicate whether each material, system or method listed is relevant to your study. If you are not sure if a list item applies to your research, read the appropriate section before selecting a response.

Materials & experimental systems

n/a	Involved in the study
☐	☒ Antibodies
☐	☒ Eukaryotic cell lines
☒	☐ Palaeontology and archaeology
☐	☒ Animals and other organisms
☒	☐ Clinical data
☒	☐ Dual use research of concern
☒	☐ Plants

Methods

n/a	Involved in the study
☒	☐ ChIP-seq
☒	☐ Flow cytometry
☒	☐ MRI-based neuroimaging

Antibodies

Antibodies used	TSPO (1:750 Novus biologicals, NB100-41398), TSPO (1:750, Abcam, ab109497), IBA1 (1:10000, Wako, 019-19741), IBA1 (1:1000, Abcam, ab48004), IBA1 (1:100, Synaptic systems, 234004), GFAP (1:500, Millipore, AB5541), HLA-DR (1:750, Invitrogen, 14-9956-82), CD68 (1:800, Fluidigm, 3159035D), GFAP (1:600, Abcam, ab218309), HLA-DR (1:750, Fluidigm, 3174025D), TSPO (1:400, Abcam, ab213654), Aβ 4G8 (1:500, Biolegend, 800702), P-TAU AT8 (1:400, ThermoFIscher, MN1020), LCP2 (1:50, Abcam, Ab196599), LCP2 (1:100, LSBiosciences, 100728), Abeta IC16 (1:400, in-house), MX04 (1:1000, Tocris 4950), p-TAU AT8 (1:400, AB223647, Invitrogen), PLP (1:200, BioRAD, MCA839G), Abeta 4G8 (1:500, Biolegend, 800702). All secondary antibodies were from Life Technologies Alexa-Fluor conjugated (A31570, A21203, A31571, A21206, A31572, A21207, A31573, A11055, A21432, A11058, A21447)
Validation	Antibody signal was positive on supplier websites for<br>TSPO (NB100-41398, MC7 cells, astrocytoma, glioblastoma and HeLA cells),<br>TSPO (ab109497, human HeLa and bladder, mouse kidney and brain),<br>IBA1 (019-19741, human and mouse brain),<br>IBA1 (ab48004, human and mouse brain),<br>IBA1 (234004, human and mouse brain and brain cultures),<br>GFAP (AB5541, human and mouse brain, neural glia and rat neonatal forebrain),<br>HLA-DR (14-9956-82, human MS brain tissue),<br>CD68 (3159035D, human PBMCs),<br>GFAP (ab218309, human cerebellum, hippocampus and mouse neural stem cells and glia),<br>HLA-DR (3174025D, human MS brain tissue),<br>TSPO (ab213654, human bladder and mouse kidney),<br>Aβ 4G8 (800702, Alzheimer's disease brain tissue),<br>P-TAU AT8 (MN1020, SH-SY5Y cells),<br>LCP2 (Ab196599, HeLA cells and Mouse spleen),<br>LCP2 (100728),<br>Abeta IC16 (in-house, human AD brain tissue, well characterised and supplied by Karsten-Korth, 10.3109/13506129.2013.797389)<br>MX04 (4950),<br>p-TAU AT8 (AB223647, SH-SY5Y cells),<br>PLP (MCA839G, human and rhesus macaque brain tissue).<br><br>All secondary antibodies have been used in previous work and are well tested by the supplier (Life Technologies). antibodies have been affinity-purified and show minimum cross-reactivity to other species.

Eukaryotic cell lines

Policy information about cell lines and Sex and Gender in Research

Cell line source(s)	BV2: Gift from Federico Roncaroli, iPSC: StemBANCC #SFC841-03-01 , EBiSC #STBCi044-A
Authentication	BV2: no authentication, iPSC: SNP analysis
Mycoplasma contamination	BV2: not tested for mycoplasma contamination, iPSC: tested negative for mycoplasma contamination
Commonly misidentified lines<br>(See ICLAC register)	none used in this study

Animals and other research organisms

Policy information about studies involving animals; ARRIVE guidelines recommended for reporting animal research, and Sex and Gender in Research

Laboratory animals	Mouse EAE: Spinal cord tissue from mice with EAE was obtained from Biozzi ABH mice housed at Queen Mary University of London, UK (originally obtained from Harlan UK Ltd, Bicester, UK). The mice were raised under pathogen-free conditions and showed a uniform health status throughout the studies. EAE was induced via injection of mouse spinal cord homogenate in complete Freund's adjuvant (CFA) into mice of 8-12 weeks or 12 months of age as. Immediately, and 24 h after injection mice were given 200ng Bordetella pertussis toxin (PT). Agematched control groups were immunized with CFA and PT.<br><br>SOD1G93A: Female hemizygous transgenic SOD1G93A mice on 129SvHsd genetic background (n=10) and corresponding non transgenic littermates (n=9) were used. This mouse line was raised at the Mario Negri Institute for Pharmacological Research-IRCCS, Milan, Italy, derived from the line (B6SJL-TgSOD1G93A-1Gur, originally purchased from Jackson Laboratories, USA) and maintained on a 129S2/SvHsd background61. The thoracic segments of spinal cord were collected from 10- and 16-week-old mice and processed.<br><br>APPNL-G-F: For the APPNL-G-F model of AD, male and female brain tissue was obtained from 11 homozygous (APPNL G F/NL-G-F) APP knock-in mice and 11 wild type mice. Mice were bred at Charles River Laboratories, UK and sampled at the Imperial College London, UK. Brain tissue samples were collected fresh from 10- and 28 week-old mice that were euthanised with sodium pentobarbital and exsanguinated.<br><br>TauP301S. Male brain tissue was obtained from 10 homozygous P301S knock-in mice and 8 wild-type C57/Bl6-OLA mice (Envigo, UK) from the Centre for Clinical Brain Sciences, Edinburgh, United Kingdom. Brain tissue samples were collected from 8- and 20-week-old mice that were perfused with PBS and 4% paraformaldehyde, with tissues being post-fixed overnight before being cryopreserved in 30% sucrose and frozen embedded in tissue tec (Leica, UK).<br><br>For all studies mice were housed 4-5 per standard cages in specific pathogen-free and controlled environmental conditions (temperature: 22±2°C; relative humidity: 55±10% and 12 h of light/dark). Food (standard pellets) and water were supplied ad libitum. Marmoset EAE: EAE was induced by subcutaneous immunization with 0.2 g of white matter homogenate emulsified in CFA in 3 adult common marmosets (Callithrix jacchus) at 4 dorsal sites adjacent to inguinal and axillary lymph nodes. Animals were monitored daily for clinical symptoms of EAE progression and assigned clinical EAE scores weekly based on extent of disability. Neurological exams were performed by a neurologist prior to each MRI scan. Animal #8 was treated with prednisolone for 5 days as part of a concurrent study (primary results not yet published). These animals were the first within their twin pair that showed three or more brain lesions by in vivo MRI and received corticosteroid treatment with the goal to reduce the severity of inflammation and potentially allow longer-term evaluation of the lesions. MRI analyses were performed according to previously published marmoset imaging protocols using T1, T2, T2*, and PD-weighted sequences on a Bruker 7T animal magnet69. Marmosets were scanned biweekly over the course of the EAE study. Following the completion of EAE studies, the brains, spinal cords, and optic nerves excised from euthanized animals were scanned by MRI for postmortem characterization of brain lesions and previously uncharacterized spinal lesions and optic nerve lesions.<br><br>TSPO-/- mice. Male and female adult TSPO-KO mice (>6 months age) and C57BL6 mice were used to test the specificity of the TSPO antibody. At the day of sacrifice, mice were anesthetized with sodium pentobarbital (200 mg/kg i.p.) and were either transcardially perfused or decapitated followed by immersion fixation in 4% paraformaldehyde for 24hrs for cryopreservation. After perfusion, the brains were extracted and post-fixed in 4% paraformaldehyde for 24 hrs. All mice were treated with LPS (1mg/kg, IP) for 3 consecutive days to increase TSPO expression.<br><br>Primary BMDM were derived from adult C57BL/6 mice after CO2 euthanization and cervical dislocation.
Wild animals	No wild animals were used in this study
Reporting on sex	no sex-based analysis have been performed, sex of animals has been reported in the supplementary tables.
Field-collected samples	No field collected samples were used in this study
Ethics oversight	Mouse EAE: Animal procedures complied with national and institutional guidelines (UK Animals Scientific Procedures Act 1986) and adhered to the 3R guidelines<br><br>SOD1G93A: Procedures involving animals and their care were conducted in conformity with the following laws, regulations, and policies governing the care and use of laboratory animals: Italian Governing Law (D.lgs 26/2014; Authorization 19/2008-A issued 6 March, 2008 by Ministry of Health); Mario Negri Institutional Regulations and Policies providing internal authorization for persons conducting animal experiments; the National Institutes of Health's Guide for the Care and Use of Laboratory Animals (2011 edition), and European Union directives and guidelines (EEC Council Directive, 2010/63/UE).<br><br>APPNL-G-F: Animal procedures complied with national and institutional guidelines (UK Animals Scientific Procedures Act 1986) and adhered to 3R guidelines.<br><br>TAUP301S: Animal procedures complied with national and institutional guidelines (UK Animals Scientific Procedures Act 1986 &

University of Edinburgh Animal Care Committees) and adhered to 3R guidelines.

Marmoset EAE: Animal procedures complied with national and institutional guidelines (NIH, Bethesda, USA)

Mouse TSPO-/- lines: Animal procedures complied with national and institutional guidelines (UK Animals Scientific Procedures Act 1986) and adhered to the 3R guidelines
BMDM donor mice: Animal experiments were approved by the Memorial University Animal Care Committee in accordance with Canadian Counsel on Animal Care guidelines

Note that full information on the approval of the study protocol must also be provided in the manuscript.
